# Brain Functional Alterations in Patients With Benign Paroxysmal Positional Vertigo Demonstrate the Visual–Vestibular Interaction and Integration

**DOI:** 10.1002/brb3.70053

**Published:** 2024-09-30

**Authors:** Jing Wu, Liang Shu, Chen‐Yan Zhou, Xiao‐Xia Du, Xu‐Hong Sun, Hui Pan, Guo‐Hong Cui, Jian‐Ren Liu, Wei Chen

**Affiliations:** ^1^ Department of Neurology Shanghai Ninth People's Hospital Shanghai Jiao Tong University School of Medicine Shanghai China; ^2^ Department of Psychology Shanghai University of Sport Shanghai China

**Keywords:** benign paroxysmal positional vertigo, occipital lobe, precuneus, resting‐state functional magnetic resonance imaging, visual–vestibular interaction

## Abstract

**Objective:**

This study aimed to analyze the features of resting‐state functional magnetic resonance imaging (rs‐fMRI) and clinical relevance in patients with benign paroxysmal positional vertigo (BPPV) that have undergone repositioning maneuvers.

**Methods:**

A total of 38 patients with BPPV who have received repositioning maneuvers and 38 matched healthy controls (HCs) were enrolled in the present study from March 2018 to August 2021. Imaging analysis software was employed for functional image preprocessing and indicator calculation, mainly including the amplitude of low‐frequency fluctuation (ALFF), fractional ALFF (fALFF), percent amplitude of fluctuation (PerAF), and seed‐based functional connectivity (FC). Statistical analysis of the various functional indicators in patients with BPPV and HCs was also conducted, and correlation analysis with clinical data was performed.

**Results:**

Patients with BPPV displayed decrease in ALFF, fALFF, and PerAF values, mainly in the bilateral occipital lobes in comparison with HCs. Additionally, their ALFF and fALFF values in the proximal vermis region of the cerebellum increased relative to HCs. The PerAF values in the bilateral paracentral lobules, the right supplementary motor area (SMA), and the left precuneus decreased in patients with BPPV and were negatively correlated with dizziness visual analog scale (VAS) scores 1 week after repositioning (W1). In addition, in the left fusiform gyrus and lingual gyrus, the PerAF values show a negative correlation with dizziness handicap inventory (DHI) scores at initial visit (W0). Seed‐based FC analysis using the seeds from differential clusters of fALFF, ALFF, and PerAF showed reductions between the left precuneus and bilateral occipital lobe, the left precuneus and left paracentral lobule, and within the occipital lobes among patients with BPPV.

**Conclusion:**

The spontaneous activity of certain brain regions in the bilateral occipital and frontoparietal lobes of patients with BPPV was reduced, whereas the activity in the cerebellar vermis was increased. Additionally, there were reductions in FC between the precuneus and occipital cortex or paracentral lobule, as well as within the occipital cortex. The functional alterations in these brain regions may be associated with the inhibitory interaction and functional integration of visual, vestibular, and sensorimotor systems. The functional alterations observed in the visual cortex and precuneus may represent adaptive responses associated with residual dizziness.

## Graphical abstract

This study discovered brain functional alterations in patients with BPPV in the visual cortex, frontoparietal lobes and cerebellar vermis that conformed to the inhibitory interaction between the multisensory systems, notably between the visual‐vestibular system. The study also emphasized the role of the precuneus for integration in BPPV. It also demonstrated the relevance of the observed brain functional alterations to severity of RD, and suggested that functional alterations in the visual cortex and precuneus were probably adaptive responses associated with RD.

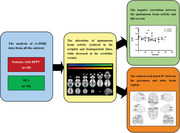

## 1 Introduction

Benign paroxysmal positional vertigo (BPPV) is a form of peripheral vestibular disorder triggered by alterations in the head orientation relative to gravity, causing intermittent vertigo and a unique type of eye movement known as nystagmus. It is responsible for up to 60% of peripheral vertigo cases (von Brevern et al. 2007). It is the most common cause of peripheral vertigo, affecting individuals aged 50–70 years old, with a higher prevalence among females. Symptoms include sudden, brief episodes of dizziness, usually not exceeding a single minute, triggered by movements such as getting up, lying down, turning over in bed, bowing, or looking up. Other potential symptoms include nausea, vomiting, heaviness in the head and feet, floating sensations, balance problems, and vibrations. The diagnosis of BPPV is mainly based on the patient's medical history and the presence of transient dizziness and characteristic nystagmus during certain head movements. Repositioning maneuver remains the most effective treatment for BPPV, and most patients experience relief from positional dizziness and nystagmus with corresponding repositioning (Shan, Peng, and Wang 2015; Tang and Li 2017). However, some patients may still experience residual dizziness (RD) with elusive mechanisms (Faralli et al. 2016; Seo et al. 2017).

The functional magnetic resonance imaging (fMRI) is a type of MRI technique that is based on the blood oxygenation level‐dependent (BOLD) effect. The resting‐state fMRI (rs‐fMRI) is a relatively new imaging technique that utilizes the spontaneous fluctuations of brain BOLD signals and combines various algorithms to evaluate the activity and connectivity levels of various brain regions, whereas the subjects are in a calm state (Bijsterbosch, Smith, and Beckmann 2017; Poldrack, Mumford, and Nichols 2011; Raimondo et al. 2021). The preprocessed rs‐fMRI data can be utilized to investigate the spontaneous activity of individual brain regions, as well as the functional connectivity (FC) between them. This can be done by using two main approaches: functional separation and functional integration.

Functional separation focuses on analyzing the activity within a single brain region, whereas functional integration looks at the correlation and mode of action among different brain regions and investigates the neural networks formed by their interconnection (Barkhof, Haller, and Rombouts 2014). To measure the level of spontaneous activity of neurons in the brain area, the amplitude of low‐frequency fluctuation (ALFF) method is used, which calculates the amplitude between 0.01 and 0.08 Hz. An increase in spontaneous activity of neurons is associated with an increase in ALFF, and vice versa (Yang et al. 2007; Zang et al. 2007). The ALFF algorithm is widely used but can be affected by noise. To address this, the fractional ALFF (fALFF) was developed, which is the ratio of ALFF to all amplitudes, thus eliminating noise signals such as cerebrospinal fluid and vascular pulsation. This standardized form of ALFF increases the accuracy of detecting spontaneous brain activity (Zou et al. 2008). The percent amplitude of fluctuation (PerAF) measures the percentage of BOLD fluctuations in comparison to the total BOLD signals at each time point and averages them over the entire time series (Jia et al. 2020). The PerAF algorithm is not as widely used as ALFF and fALFF, and more research is needed to explore its stability and reliability in diseases.

Seed‐based FC analysis and independent component analysis (ICA) are examples of functional integration methods (Li et al. 2009; Smitha et al. 2017; van den Heuvel and Hulshoff Pol 2010). The seed‐based FC analysis approach includes voxel‐wise FC and region‐of‐interest (ROI)‐wise FC analysis. The primary distinction between the two approaches is that voxel‐wise FC analysis focuses on the functional connections between seeds and various voxels in the whole brain, whereas ROI‐wise FC analysis focuses on the connections between various ROIs (Biswal et al. 1995; Cole, Smith, and Beckmann 2010).

Due to the uneven input from the bilateral otoliths after BPPV episodes and repositioning, it may lead to adaptive changes in the central vestibular system and associated brain areas. There is limited research on fMRI in relation to BPPV or RD, and the exact nature of this adaptive alteration is still unknown. Moreover, it is yet to be determined if the functional alterations in the central vestibular system are linked to the cause of RD. The main objective of this study is to delve into this issue.

## 2 Materials and Methods

### 2.1 Subjects

This study was conducted to prospectively analyze rs‐fMRI data from 38 patients with BPPV who visited the Vertigo Clinic in our hospital between March 2018 and August 2021. Selection criteria for research subjects included patients aged between 18 and 80 years old who met the diagnostic criteria for BPPV in the 2017 Guidelines for Diagnosis and Treatment and had unilateral posterior semicircular canal BPPV or horizontal semicircular canal BPPV. Additionally, repositioning was successful at initial visit (W0) and Dix‐Hallpike, and supine roll tests were repeated with no dizziness or nystagmus at 1 week after repositioning (W1). Excluded from the research were those with a prior history of severe diseases or organ dysfunction, such as sudden deafness, Meniere's disease, vestibular neuritis (VN), severe traumatic brain injury, cerebrovascular disease, epilepsy, drug dependence, schizophrenia, vestibular migraine, central positional vertigo, multiple semicircular canal involvement in BPPV, softening lesions, white matter degeneration, or space‐occupying lesions on cranial MRI. Additionally, data were collected from HCs matched with gender and age during the same period.

### 2.2 Clinical Parameters of Subjects

The demographic data of all participants were obtained. For patients with BPPV, duration for repositioning and fMRI scanning and affected semicircular canal were also documented at W0. To evaluate the severity of RD, the vertigo visual analog scale (VAS) and dizziness VAS are commonly employed, with scores ranging from 0 (no symptoms) to 100 (very severe symptoms) (Toupet, Ferrary, and Grayeli 2011). Furthermore, the dizziness handicap inventory (DHI) questionnaire is commonly utilized to assess patients’ quality of life, with a total score ranging from 0 to 100 points. A higher score indicates a more significant impact of the disease on patients’ quality of life (Chen et al. 2016; Lee, Kwon, and Ban 2009).

### 2.3 Data Acquisition

The Shanghai Key Laboratory of Magnetic Resonance at East China Normal University conducted an MRI data collection of resting‐state functional and structural magnetic resonance images using a Siemens Prisma 3.0 T instrument with a 64‐channel head coil. Prior to the scan, a device was used to immobilize the head to minimize head movement. Subjects were instructed to remain calm with their eyes closed during the functional imaging scan. The *T*2*‐weighted planar gradient echo sequence was employed for the functional imaging scan, with parameters of TR 2000 ms, TE 30 ms, reverse angle 90°, 33 layers with layer thickness of 3.5 mm, field of view of 220 mm × 220 mm, 64 × 64 acquisition matrix, 240 time points, and layer spacing of 25%. The *T*1‐weighted 3D magnetic resonance sequence was used for high‐resolution structural imaging, with parameters of TR 2530 ms, TE 2.34 ms, inversion time 1100 ms, inversion angle 7°, 192 layers with a layer thickness of 1 mm, field of view of 256 mm × 256 mm, and a 256 × 256 acquisition matrix.

### 2.4 Data Preprocessing

The raw resting‐state data were preprocessed using the RESTplus v1.25 software (http://www.restfmri.net/) on the MATLAB R2013b platform. The steps included: (1) converting the DICOM data into the NIFTI format; (2) discarding the first 10 time points; (3) temporal alignment; (4) head motion correction, with subjects with more than 3 mm of translational and rotational movement being excluded; (5) co‐registering the *T*1‐weighted images and functional images with the previous links as the origin; (6) using the DARTEL registration method to register the *T*1‐weighted images and the corresponding functional images; (7) applying a half‐height full‐width Gaussian filter for spatial smoothing with an FWHM of 6 mm; (8) removing linear drift; (9) using multiple linear regression to remove the effects of 24 head movement parameters, cerebrospinal fluid, and white matter signals on the BOLD signals; (10) filtering (0.01 < *f* < 0.08 Hz) to reduce low and high‐frequency noise (ALFF, fALFF, and PerAF did not perform this step).

### 2.5 Data Processing and Analysis

Utilizing RESTplus v1.25 for data processing of functional images, 3D statistical parameter maps of patients and healthy controls are obtained. ALFF, fALFF, and PerAF are calculated as indicators, and the signal values of each voxel are divided by the average of the whole brain voxel ALFF, fALFF, and PerAF to reduce errors and standardize, thus producing mALFF and mfALFF, and mPerAF for further statistical analysis.

SPM12 (http://www.fil.ion.ucl.ac.uk/spm/) was used to a conduct two‐sample *t*‐test on the statistical maps of ALFF, fALFF, and PerAF for two groups of subjects, with age and gender as covariates for regression. The *t*‐test results were adjusted for multiple comparisons using the cluster‐level family‐wise error (FWE) correction (voxel‐level *p* value <0.001, cluster‐level *p* value <0.05), and the surviving brain areas were identified as differential brain regions. XjView (https://www.alivelearn.net/xjview/) was used to report the results of the differential brain regions, listing the AAL brain regions according to peak *T* value. Visualization was done using MRIcroGL (https://www.nitrc.org/plugins/mwiki/index.php/metrirogl). Using RESTplus, the values of mALFF, mfALFF, and mPerAF were extracted from the brain regions of the patients and HCs, and statistical charts were created in GraphPad Prism software.

Seed‐based FC analysis was conducted using RESTplus to calculate voxel‐wise FC and generate voxel‐level whole brain FC maps. The *z* value was utilized for subsequent statistical analysis. The differential brain regions identified by functional indicators of fALFF, ALFF, and PerAF were used as seed points for FC calculations. Using SPM12, a two‐sample *t*‐test was performed on the FC statistical maps, with age and gender included as covariates for regression. Distinct brain areas were reported using xjView, and visualization was done using MRIcroGL and BrainNet Viewer v1.7.

### 2.6 Statistical Analysis

Statistical analysis of the demographic and clinical data for two groups of subjects was done using SPSS 26.0 software. Numerical variables with normal distribution were represented by mean ± standard deviation, and numerical variables with skewed distribution were described by median (25th–75th percentile). Mann–Whitney *U*‐test was applied on numerical variables, and chi‐square test or Fisher's exact test was conducted on categorical variables. Two‐sample *t*‐test on the statistical maps of ALFF, fALFF, PerAF, and FC for two groups of subjects was done by utilizing SPM12. Utilizing SPSS, we conducted a correlational analysis between the functional indicators and clinical data, including dizziness VAS and DHI. The Pearson correlation coefficient was employed for normally distributed data, whereas the Spearman correlation coefficient was used for non‐normally distributed data.

## 3 Results

### 3.1 Demographic and Clinical Information of Subjects

Demographic and clinical characteristics of patients with BPPV and HCs are shown in Table 1. Patients with BPPV with dizziness VAS score >10 after repositioning were labeled having RD, whereas those with dizziness VAS score ≤10 were labeled not having RD.

**TABLE 1:** The demographic and clinical characteristics of patients with benign paroxysmal positional vertigo (BPPV) and HCs.

	BPPV patients (*n* = 38)	BPPV with RD (*n* = 29)	BPPV without RD (*n* = 9)	HCs (*n* = 38)	*P*_1_	*P*_2_
Gender (male/female)	10/28	7/22	3/6	12/26	0.613	0.673
Age (years)	59.5 (48.25–63.25)	59 (46.5–65.5)	61 (48–61.5)	58 (48.25–63.25)	0.967	0.877
Duration for repositioning (days)	7 (4.75–14)	7 (4–12.5)	10 (7–21)	/	/	0.134
Duration for fMRI scanning (days)	17.5 (13–34.25)	21 (12–46.5)	17 (14–30)	/	/	0.850
Affected SCC (left/right)	11/27	9/20	2/7	/	/	1.000
Affected SCC (PC/HC)	29/9	22/7	7/2	/	/	1.000
DHI score at W0	33.89 ± 15.37	37.72 ± 14.06	16 (12–27)	/	/	0.005**
Dizziness VAS score at W1	31.97 ± 22.94	41.21 ± 17.81	0 (0–5)	/	/	<0.001***

*Note*: W1, 1 week after repositioning; W0, baseline (initial visit); *P*_1_, a comparison between patients with BPPV and HCs, *P*_2_, a comparison between the patients with RD and those without RD.

Abbreviations: DHI, dizziness handicap inventory; fMRI, functional magnetic resonance imaging; HC, horizontal semicircular canal; PC, posterior semicircular canal; RD, residual dizziness; SCC, semicircular canal; VAS, visual analog scale.

^**^ *p* < 0.01.

^***^ *p* < 0.001.

### 3.2 Fractional Amplitude of Low‐Frequency Fluctuation

**FIGURE 1:**
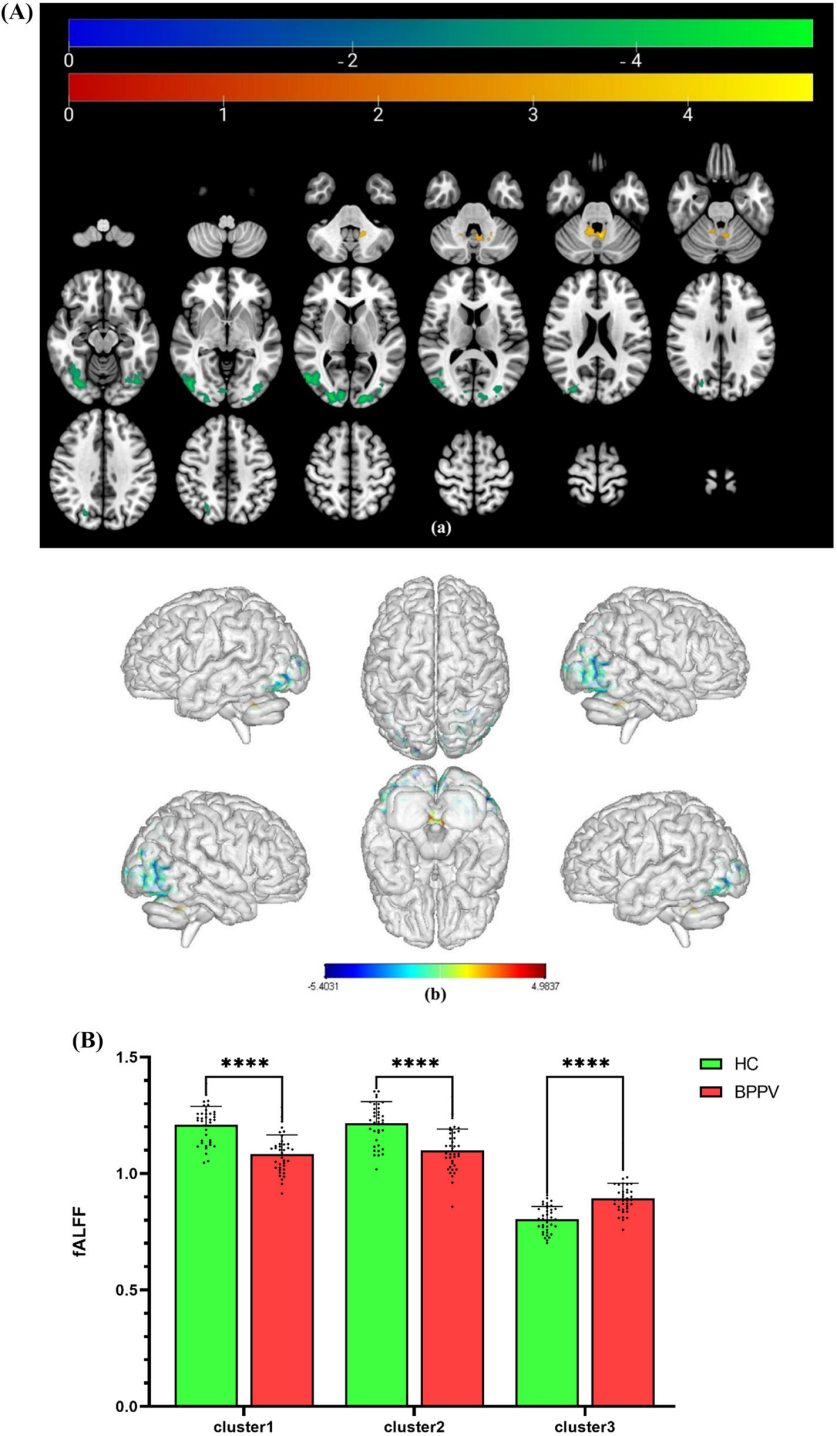
(A) The brain regions where the fALFF values differ between patients with BPPV and HCs. Warm color tones signify brain regions with higher fALFF values in patients with BPPV compared to HCs, whereas cold color tones represent brain regions with lower fALFF values in patients with BPPV in comparison to HCs. The color bar indicates the *T* value, (a and b) illustrate the brain regions showing significant differences, and the following images are the same. (B) The comparison of fALFF values between patients with BPPV and HCs. In comparison to HCs, the fALFF values of patients with BPPV in Clusters 1 and 2 were significantly decreased, whereas significantly increased in Cluster 3, **** stands for *p* < 0.0001. BPPV, benign paroxysmal positional vertigo; fALFF, fractional amplitude of low frequency fluctuation.

The brain regions that showed significant differences between the two groups were mainly located in the bilateral occipital lobe and cerebellar vermis. Patients with BPPV had a decreased fALFF in the right superior occipital gyrus (*x* = 21, *y* = −93, *z* = 3, *t* = −5.4031) and the left middle occipital gyrus (*x* = −15, *y* = −93, *z* = 6, *t* = −5.0457), with a voxel‐level corrected threshold of *p* < 0.001 and a cluster‐level FWE corrected threshold of *p* < 0.05, cluster size greater than 237 voxels. In contrast, the vermis of the cerebellum (*x* = −3, *y* = −54, *z* = −30, *t* = 4.9837) and the anterior cerebellar lobe (*x* = −12, *y* = −45, *z* = −36, *t* = 4.62) had a significantly increased fALFF, voxel‐level corrected threshold *p* < 0.001, cluster‐level FWE corrected threshold *p* < 0.05, and cluster size greater than 69 voxels, as illustrated in Figure 1A and Table 2. The comparison of fALFF values between the two groups is depicted in Figure 1B.

**TABLE 2:** The brain regions where the fractional amplitude of low‐frequency fluctuation (fALFF) values differ between patients with benign paroxysmal positional vertigo (BPPV) and HCs.

Comparison of fALFF	Predominant regions in each cluster	Cluster size (voxels)	MNI coordinates			Peak *T* value	Cluster‐level FWE correction voxel‐level *p* value/cluster‐level *p* value
			*x*	*y*	*z*		
**BPPV < HC**	**Cluster 1**	**519**					**<0.001/<0.05**
	Occipital_Sup_R	47	21	−93	3	−5.40	
	Occipital_Mid_R	94	45	−78	0	−5.05	
	Temporal_Mid_R	91	45	−72	0	−4.95	
	Fusiform_R	74	30	−75	−12	−4.68	
	Calcarine_R	57	24	−57	15	−4.08	
	Occipital_Inf_R	52	42	−81	−6	−3.84	
	**Cluster 2**	**237**					**<0.001/<0.05**
	Occipital_Mid_L	95	−15	−93	6	−5.05	
	Occipital_Inf_L	62	−42	−72	−12	−4.75	
	Occipital_Sup_L	26	−12	−93	9	−4.03	
	Fusiform_L	19	−24	−45	−12	−3.60	
**BPPV > HC**	**Cluster 3**	**69**					**<0.001/<0.05**
	Vermis_9	7	−3	−54	−30	4.98	
	Cerebellum anterior lobe	58	−12	−45	−36	4.62	

*Note*: “Occipital_Sup_R” refers to the right superior occipital gyrus in the AAL template, with comparable abbreviations applying to the other regions. The bold text signifies the inclusion and precedence.

Abbreviations: FWE, family‐wise error; Inf, inferior; L, left; Mid, middle; MNI, montreal neurological institute; R, right; Sup, superior.

### 3.3 Amplitude of Low‐Frequency Fluctuation

**FIGURE 2:**
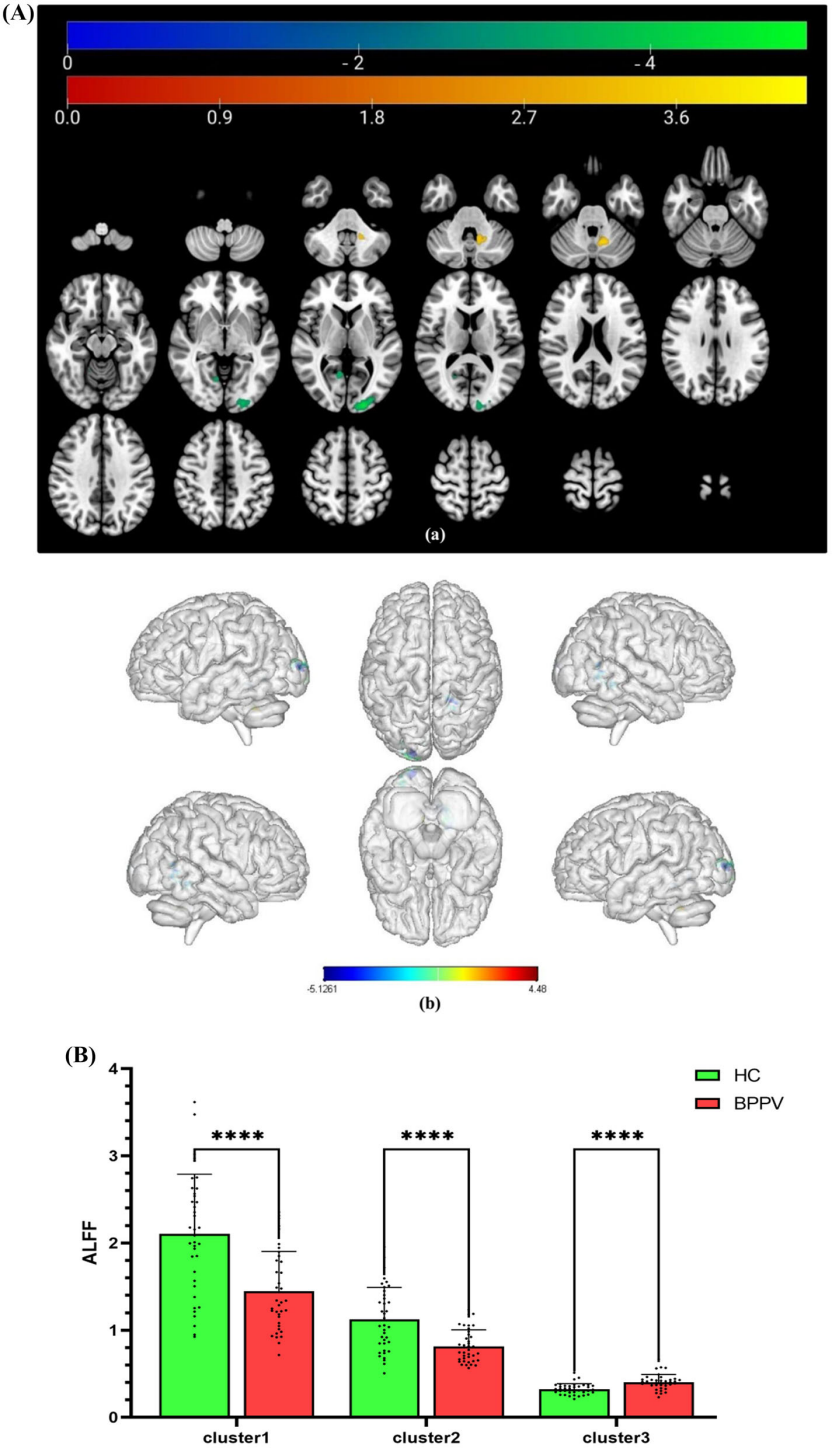
(A) The brain regions where the ALFF values differ between patients with BPPV and HCs. Warm color tones signify brain regions with higher ALFF values in patients with BPPV compared to HCs, whereas cold color tones represent brain regions with lower ALFF values in patients with BPPV in comparison to HCs. (B) The comparison of ALFF values between patients with BPPV and HCs. In comparison to HCs, the ALFF values of patients with BPPV in Clusters 1 and 2 were significantly decreased, whereas significantly increased in Cluster 3, **** stands for *p* < 0.0001. ALFF, amplitude of low frequency fluctuation; BPPV, benign paroxysmal positional vertigo.

The brain regions with differences between the two groups were located in the bilateral occipital lobe and the proximal vermis of the left cerebellum. Compared to HCs, patients with BPPV showed a decreased ALFF in the left middle occipital gyrus (*x* = −18, *y* = −96, *z* = 6, *t* = −5.1261) and the right lingual gyrus (*x* = 24, *y* = −45, *z* = −9, *t* = −4.2132), with a voxel‐level corrected threshold *p* < 0.001, cluster‐level FWE corrected threshold *p* < 0.05, and cluster size greater than 75 voxels. On the other hand, there was an increased ALFF mainly in the left cerebellar tonsil (*x* = −18, *y* = −51, *z* = −39, *t* = 4.48), voxel‐level corrected threshold *p* < 0.001, cluster‐level FWE corrected threshold *p* < 0.05, and cluster size greater than 43 voxels, as depicted in Figure 2A and Table 3. Figure 2B displays the comparison of ALFF values between the two groups.

**TABLE 3:** The brain regions where the amplitude of low frequency fluctuation (ALFF) values differ between patients with benign paroxysmal positional vertigo (BPPV) and HCs.

Comparison of ALFF	Predominant regions in each cluster	Cluster size (voxels)	MNI coordinates			Peak *T* value	Cluster‐level FWE correction voxel‐level *p* value/cluster‐level *p* value
			*x*	*Y*	*z*		
**BPPV < HC**	**Cluster 1**	**128**					**<0.001/<0.05**
	Occipital_Mid_L	84	−18	−96	6	−5.13	
	Occipital_Sup_L	23	−12	−96	9	−4.13	
	Calcarine_L	4	−12	−63	6	−4.13	
	Occipital_Inf_L	13	−21	−93	−3	−4.03	
	**Cluster 2**	**75**					**<0.001/<0.05**
	Lingual_R	52	24	−45	−9	−4.21	
	Fusiform_R	8	24	−66	−9	−3.99	
	Calcarine_R	9	18	−90	3	−3.98	
**BPPV > HC**	**Cluster 3**	**43**					**<0.001/<0.05**
	Cerebellar tonsil	6	−18	−51	−39	4.48	
	Cerebellum anterior lobe	35	−12	−57	−33	4.32	

*Note*: The bold text signifies the inclusion and precedence.

Abbreviation: FWE, family‐wise error.

### 3.4 Percent Amplitude of Fluctuation

**FIGURE 3:**
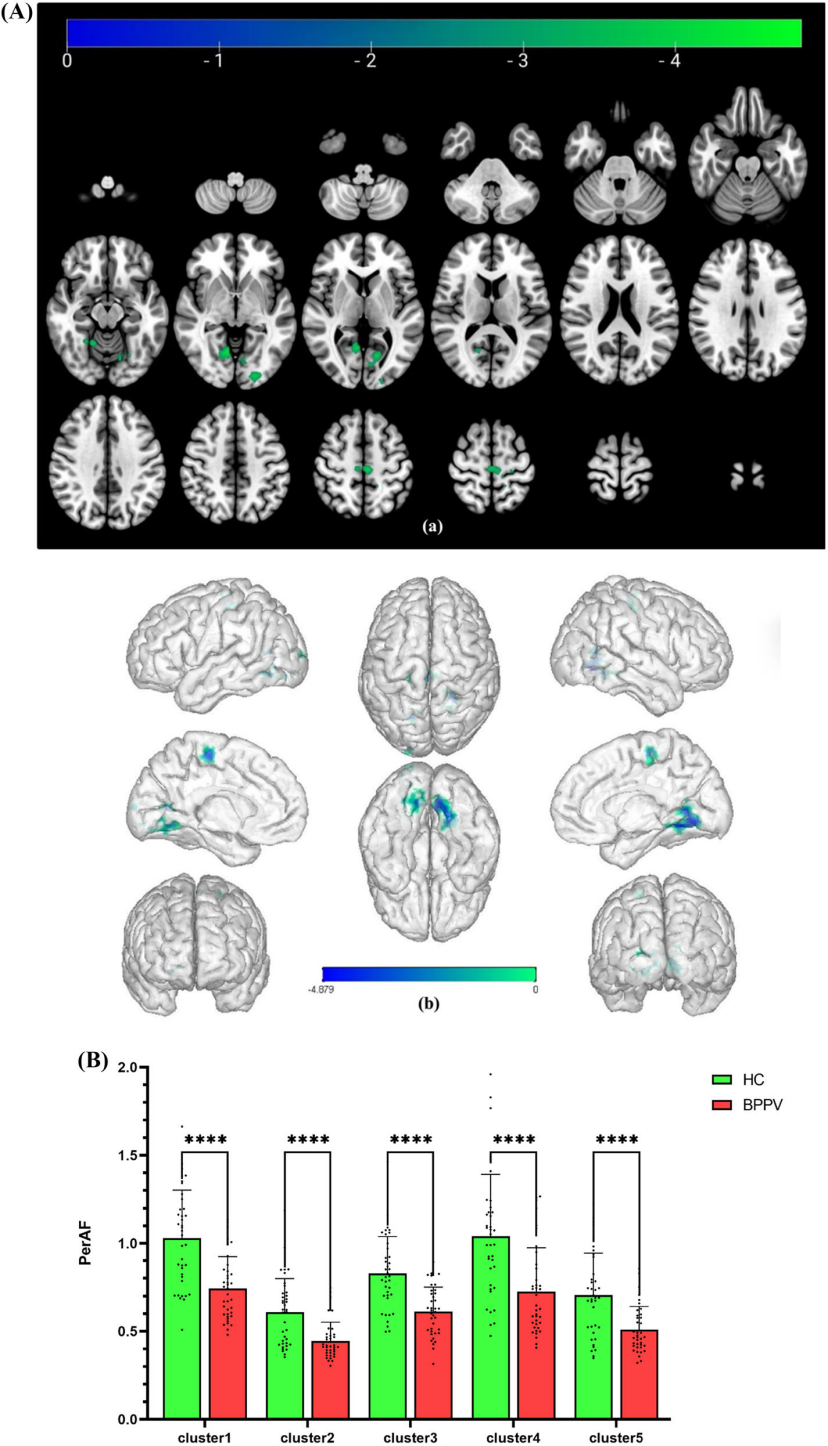
(A) The brain regions where the PerAF values differ between patients with BPPV and HCs. Cold color tones represent brain regions with lower PerAF values in patients with BPPV in comparison to HCs, and the following images are the same. (B) The comparison of PerAF values between patients with BPPV and HCs. In comparison to HCs, the PerAF values of patients with BPPV in Clusters 1–5 were significantly decreased, **** stands for *p* < 0.0001. BPPV, benign paroxysmal positional vertigo; PerAF, percent amplitude of fluctuation.

The brain regions that showed significant differences between the two groups were mainly concentrated in the bilateral occipital and frontoparietal lobes. Compared to HCs, patients with BPPV had decreased PerAF mainly in the right lingual gyrus (*x* = 12, *y* = −63, *z* = −6, *t* = −4.879), the left paracentral lobule (*x* = −6, *y* = −24, *z* = 57, *t* = −4.4625), the left fusiform gyrus (*x* = −30, *y* = −63, *z* = −6, *t* = −4.3731), the left calcarine (*x* = −12, *y* = −63, *z* = 9, *t* = −4.1377), the left inferior occipital gyrus (*x* = −21, *y* = −90, *z* = −6, *t* = −4.0551), voxel‐level corrected threshold *p* < 0.001, cluster‐level FWE corrected threshold *p* < 0.05, and cluster size greater than 50 voxels. This is demonstrated in Figure 3A and Table 4. Figure 3B shows the comparison of PerAF values between the two groups.

**TABLE 4:** The brain regions where the percent amplitude of fluctuation (PerAF) values differ between patients with benign paroxysmal positional vertigo (BPPV) and HCs.

Comparison of PerAF	Predominant regions in each cluster	Cluster size (voxels)	MNI coordinates			Peak *T* value	Cluster‐level FWE correction voxel‐level *p* value/cluster‐level *p* value
			*x*	*y*	*z*		
**BPPV < HC**	**Cluster 1**	**146**					**<0.001/<0.05**
	Lingual_R	98	12	−63	−6	−4.88	
	Fusiform_R	12	27	−48	−9	−4.28	
	Calcarine_R	19	18	−87	3	−3.99	
	**Cluster 2**	**79**					**<0.001/<0.05**
	Paracentral_Lobule_L	47	−6	−24	57	−4.46	
	Supp_Motor_Area_R	15	3	−21	60	−3.69	
	Precuneus_L	4	−15	−42	57	−3.67	
	Paracentral_Lobule_R	2	9	−27	69	−3.47	
	**Cluster 3**	**57**					**<0.001/<0.05**
	Fusiform_L	14	−30	−63	−6	−4.37	
	Lingual_L	30	−15	−60	−9	−4.30	
	Cerebellum_6_L	12	−18	−66	−15	−3.59	
	**Cluster 4**	**53**					**<0.001/<0.05**
	Calcarine_L	32	−12	−63	9	−4.14	
	Lingual_L	21	−9	−75	3	−3.66	
	**Cluster 5**	**50**					**<0.001/<0.05**
	Occipital_Inf_L	20	−21	−90	−6	−4.06	
	Occipital_Mid_L	17	−18	−96	6	−3.54	
	Fusiform_L	6	−24	−81	−9	−3.49	
	Lingual_L	6	−18	−84	−9	−3.44	

*Note*: The bold text signifies the inclusion and precedence.

Abbreviations: FWE, family‐wise error; Supp_Motor_Area, supplementary motor area.

### 3.5 The Correlation Between Dizziness VAS Score at W1 and PerAF

**FIGURE 4:**
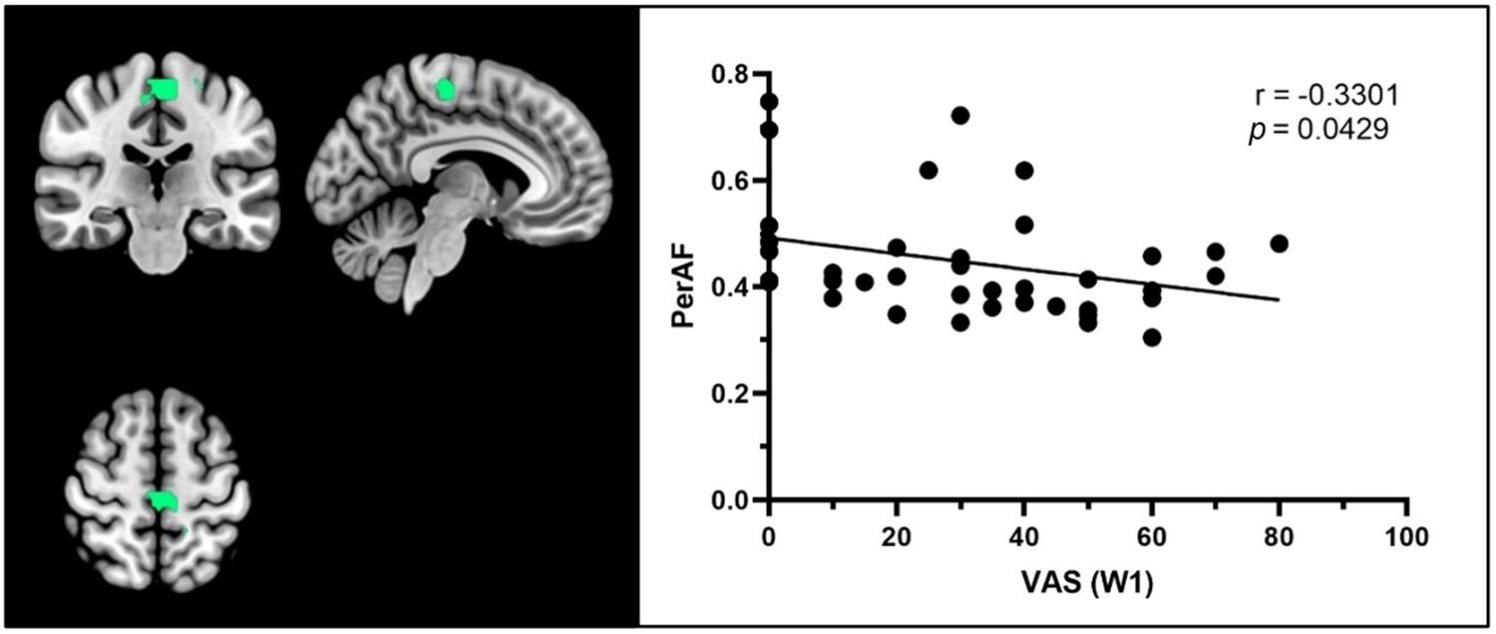
The correlation between PerAF values in Cluster 2 (mainly located in the bilateral paracentral lobules, right supplementary motor area [SMA], and left precuneus) and dizziness VAS score at W1. PerAF, percent amplitude of fluctuation; VAS, visual analog scale.

Through SPSS correlation analysis, it was observed that the dizziness VAS score at W1 was inversely correlated with the PerAF value of Cluster 2 (mainly located in the bilateral paracentral lobules, right supplementary motor area (SMA), and left precuneus) of the differential brain regions where the PerAF values differed between patients with BPPV and HCs. The Spearman correlation coefficient was −0.3301, as shown in Figure 4; there was no significant correlation between the dizziness VAS score or duration and other functional indicators of the differential brain regions between the two groups.

### 3.6 The Correlation Between DHI Score at W0 and PerAF

**FIGURE 5:**
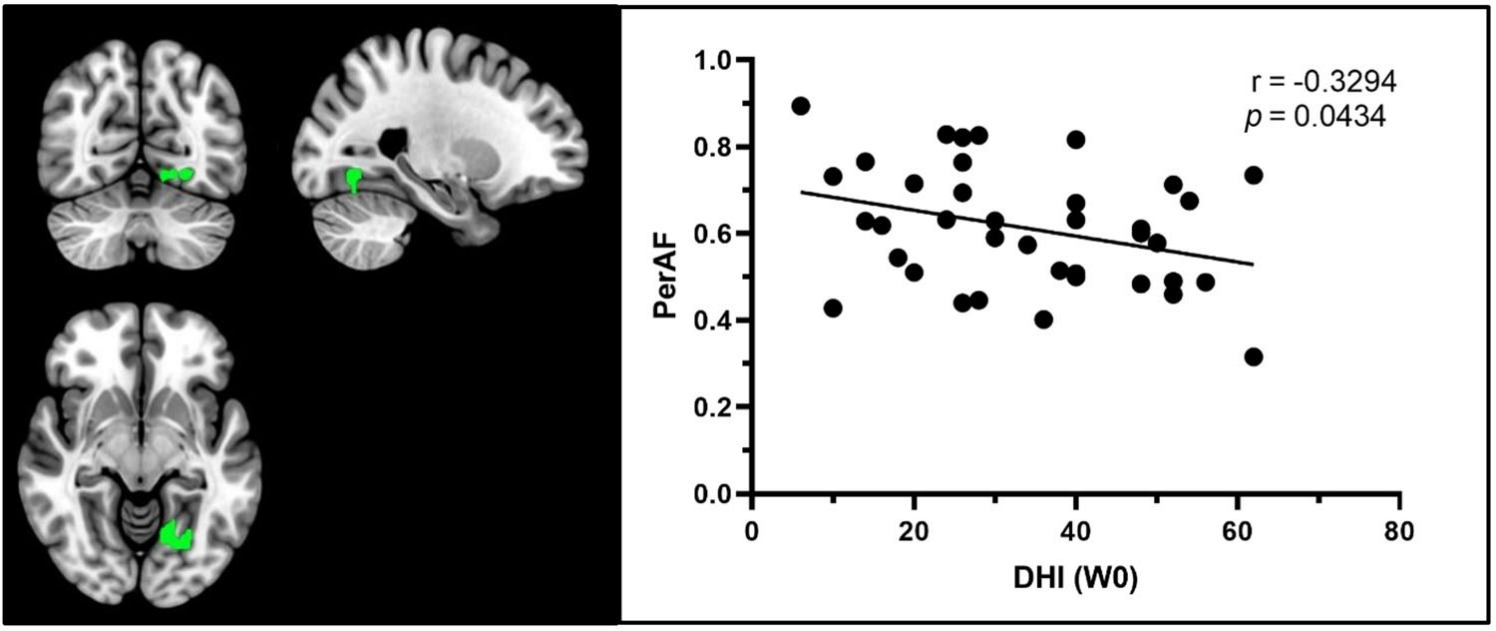
The correlation between PerAF values in Cluster 3 (mainly located in the left fusiform and lingual gyrus) and DHI score at W0. DHI, dizziness handicap inventory; PerAF, percent amplitude of fluctuation.

Upon performing SPSS correlation analysis, it was evident that a disparity existed in the DHI score at W0 and the PerAF value between patients with BPPV and HCs. Notably, in the brain region, the PerAF value of Cluster 3 (predominantly located in the left fusiform gyrus and left lingual gyrus) displayed a negative correlation, with a Pearson correlation coefficient of −0.3294, as illustrated in Figure 5. Conversely, there was no notable correlation between the DHI score and other functional indicators of the two groups with differential brain regions.

### 3.7 Seed‐Based FC Analysis

The FC of brain regions was assessed using fALFF, ALFF, and PerAF as seeds: (1) When using the differential Cluster 1 of fALFF (mainly located in the right superior occipital and middle occipital gyrus) as seeds, the FC values mainly in the left precuneus of patients with BPPV were found to be significantly reduced compared to HCs. (2) When using the differential Cluster 1 of ALFF (mainly located in the left middle occipital gyrus) as the seed, patients with BPPV exhibited significantly reduced FC values in the left middle occipital, right inferior occipital, and right middle occipital gyrus. When Cluster 2 of ALFF (mainly in the right lingual gyrus) was used as the seed, the FC values significantly decreased mainly in the right superior occipital gyrus and right cuneus. (3) Additionally, using Cluster 2 of PerAF (mainly situated in the left paracentral lobule) as the seed resulted in decreased FC values in the left precuneus, left paracentral lobule, and right postcentral gyrus. Furthermore, using Cluster 3 of PerAF (mainly in the left lingual gyrus) as the seed led to a decrease in FC values in the left precuneus. The Cluster 1 of PerAF (mainly located in the right lingual gyrus) and Cluster 5 (mainly in the left inferior occipital and middle occipital gyrus) highly overlapped with Clusters 2 and 1 of ALFF, as a result, the findings of FC are omitted. There was no notable change in FC when utilizing the other differential clusters of fALFF, ALFF, and PerAF as seed. For these results, the voxel‐level correction threshold was *p* < 0.001, with a cluster‐level FWE correction threshold of *p* < 0.05. Refer to Figure 6A,B and Table 5 for more details. This comparison of FC values between the two groups is depicted in Figure 6C.

FIGURE 6(A) The brain regions where FC values of patients with BPPV were significantly reduced compared to HCs. The results obtained by using fALFF, PerAF, ALFF differential clusters as seed were represented by dark blue, blue, and green, respectively. (B) The FC reductions between brain regions in patients with BPPV. The blue connecting lines in a three‐dimensional brain map from various orientations indicate FC reductions between diverse brain regions in patients with BPPV. LING.L, left lingual gyrus; LING.R, right lingual gyrus; PCL.L, left paracentral lobule; PCUN.L, left precuneus; SOG.R, right superior occipital gyrus. (C) The comparison of FC values between patients with BPPV and HCs. FC values of patients with BPPV in Clusters 1–5 were significantly decreased than HCs, **** stands for *p* < 0.0001. BPPV, benign paroxysmal positional vertigo; FC, functional connectivity.

**Figure:**
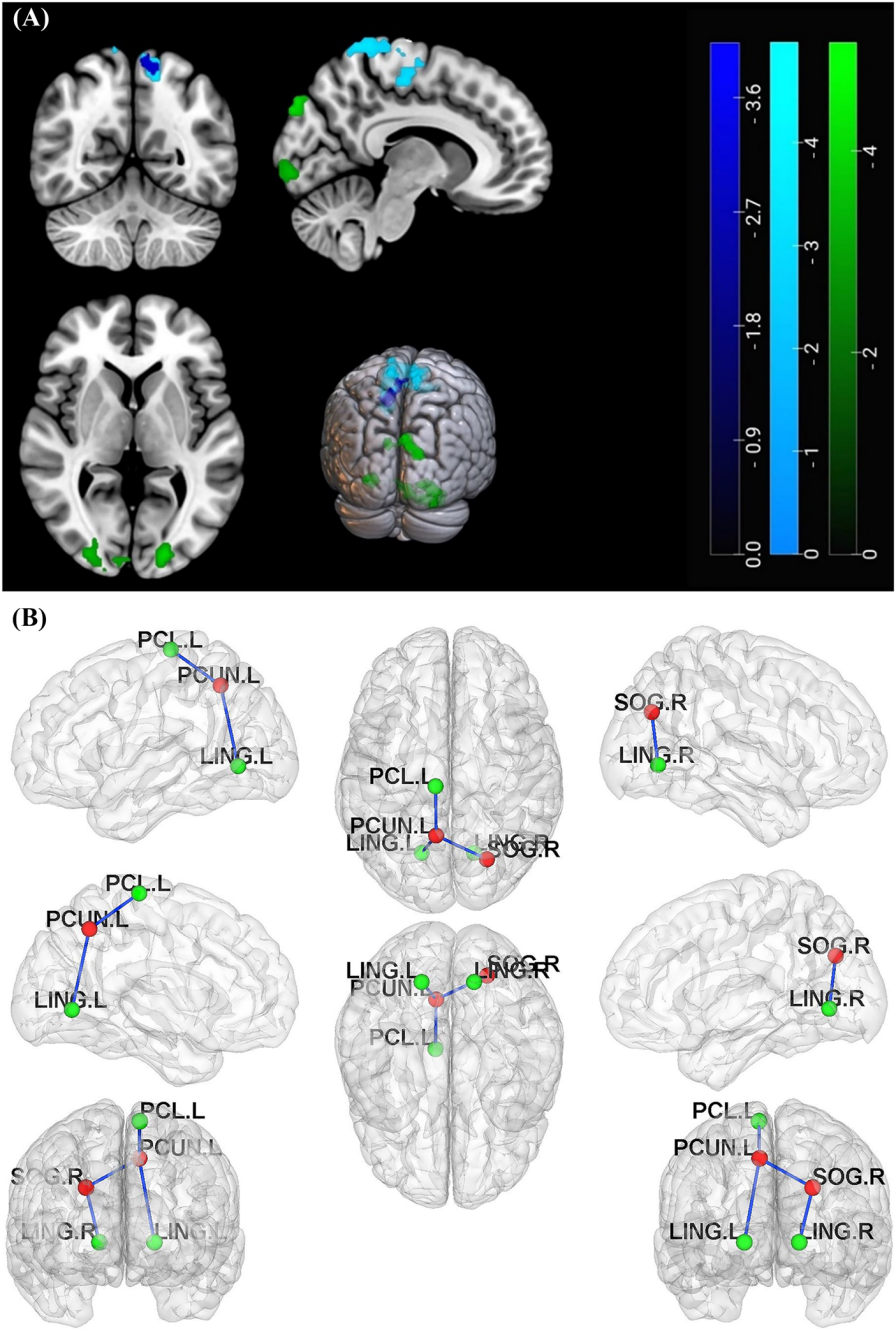

**Figure:**
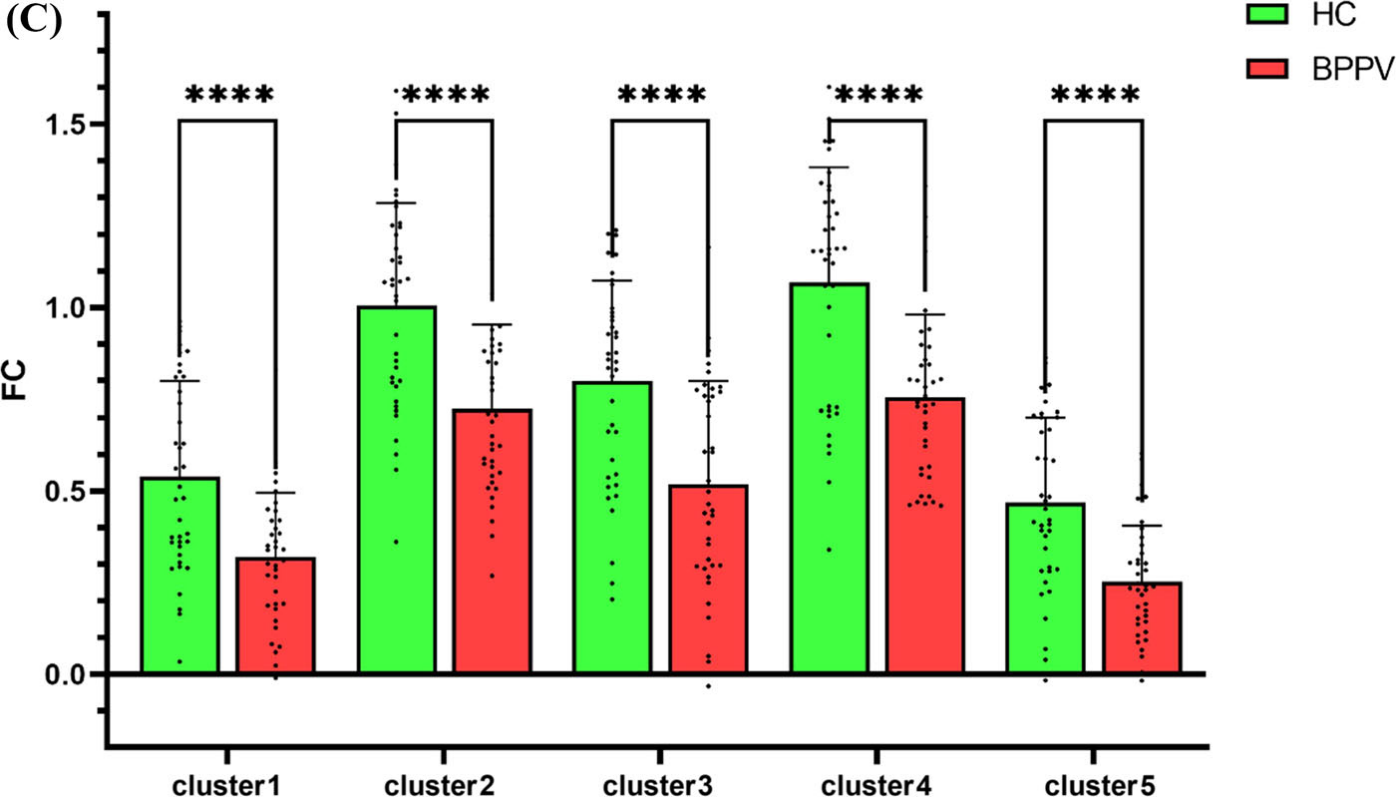

**TABLE 5:** The brain regions where functional connectivity (FC) values of patients with benign paroxysmal positional vertigo (BPPV) were significantly reduced compared to HCs.

Indicators seeds are based on	Seeds	Predominant regions in each cluster	Cluster size (voxels)	MNI coordinates			Peak *T* value	Cluster‐level FWE correction voxel‐level *p* value/cluster‐level *p* value
				*x*	*y*	*z*		
fALFF	**Cluster 1 (mainly in Occipital_Sup_R, Occipital_Mid_R)**	**Cluster 1**	**57**					**<0.001/<0.05**
		Precuneus_L	50	−12	−60	60	−4.07	
ALFF	**Cluster 1 (mainly in Occipital_Mid_L)**	**Cluster 2**	**267**					**<0.001/<0.05**
		Occipital_Mid_L	40	−18	−93	6	−5.28	
		Occipital_Inf_R	48	30	−87	−12	−4.28	
		Occipital_Mid_R	22	30	−90	0	−3.97	
	**Cluster 2 (mainly in Lingual_R)**	**Cluster 3**	**80**					**<0.001/<0.05**
		Occipital_Sup_R	28	18	−90	30	−4.36	
		Cuneus_R	27	9	−90	36	−4.10	
		Precuneus_R	13	6	−84	42	−4.08	
PerAF	**Cluster 2 (mainly in Paracentral_Lobule_L)**	**Cluster 4**	**379**					**<0.001/<0.05**
		Precuneus_L	45	−12	−45	75	−5.00	
		Paracentral_Lobule_L	105	−9	−36	69	−4.81	
		Postcentral_R	35	12	−51	75	−4.71	
	**Cluster 3 (mainly in Lingual_L)**	**Cluster 5**	**110**					**<0.001/<0.05**
		Precuneus_L	65	−12	−57	60	−4.35	

*Note*: The bold text signifies the inclusion and precedence.

Abbreviations: ALFF, amplitude of low frequency fluctuation; fALFF, fractional amplitude of low frequency fluctuation; FWE, family‐wise error; PerAF, percent amplitude of fluctuation.

## 4 Discussion

The correlation between dizziness and multiple brain regions has been established by the results of various neuroimaging studies (Varangot‐Reille et al. 2022). However, there is a scarcity of fMRI research on the neural activity of patients with BPPV in the brain area. Fu et al. (2022) discovered, through rs‐fMRI, that those patients with BPPV who experienced RD after repositioning had significantly lower ALFF in the bilateral precuneus when compared to those without RD. Our team's prior analysis of rs‐fMRI focused on the cerebellum and brainstem with a smaller sample size revealed that patients with BPPV post‐repositioning had a heightened fALFF in pontine and ReHo values in the left cerebellum (posterior lobe/Crus II), implying adaptive changes in the central vestibular system of these patients (Zhu et al. 2021). Adaptive changes in the central vestibular system and other related brain areas may arise due to the unequal input from the bilateral otoliths before and after repositioning. How do the brain function alterations manifest in patients with BPPV, and what is the connection between these alterations and the prevalent RD after repositioning? Our study contributes additional findings to this issue.

### 4.1 The Brain Function Alterations in Patients With BPPV

#### 4.1.1 The Spontaneous Activity Was Reduced in the Visual Cortex

Our research has shown that patients with BPPV exhibit less spontaneous activity in the bilateral occipital lobes of the brain compared to HCs. The occipital lobe is responsible for processing visual information, and any disruption to its functioning or structure can lead to visual impairment (Fraser, Newman, and Biousse 2011; Lee et al. 2001). The visual cortex comprises two main regions: the striate cortex (V1) and the extrastriate visual areas (V2–V4, etc.). V1, also known as the primary visual cortex, includes the Brodmann 17 area situated around the calcarine sulcus and receives input from the lateral geniculate body. The extrastriate areas consist of Brodmann 18 and 19 regions, which make up the higher visual cortex (Chauhan et al. 2021; Palejwala et al. 2021). The lingual gyrus and cuneus, situated on the inner side of the occipital lobe, housing the functional regions of V1–V4, are crucial visual central structures and are involved in visual information processing (Palejwala et al. 2021). The lateral cortex of the occipital lobe contains the superior, middle, and inferior gyrus, which play roles in object recognition, facial recognition, and motion perception (Palejwala et al. 2020; Wei et al. 2023). The fusiform gyrus in the ventral temporal lobe is crucial for advanced visual functions like facial perception, object recognition, and reading (Weiner and Zilles 2016). Our study also found decreased spontaneous activity in the fusiform gyrus and middle temporal gyrus, with the middle temporal gyrus also being involved in visual information processing (Leshinskaya and Thompson‐Schill 2020; Vannini et al. 2008). Moreover, there was a reduction in the fALFF values in the right middle temporal gyrus, which plays a role in analyzing visual inputs (Leshinskaya and Thompson‐Schill 2020; Vannini et al. 2008). The integration of multiple sensory systems (vestibular, visual, and proprioceptive) is necessary to perceive movement, spatial positioning, eye movement, and posture control of the human body (Cutfield et al. 2014). The collaboration between these three systems can prevent a feeling of imbalance (Della‐Justina et al. 2015). In studies of vertigo‐related diseases, structures related to visual pathways have often been observed to have a deactivation pattern (Varangot‐Reille et al. 2022). Bense et al. (2004) conducted a positron emission tomography (PET) study on patients with unilateral peripheral vestibular lesions caused by VN and found that during the acute phase, glucose metabolism in various sensory vestibular cortex and subcortical areas (including the posterior temporal vestibular cortex, posterior lateral thalamic area, anterior cingulate gyrus, brainstem, and hippocampus) increased significantly, whereas, in the visual cortex, the glucose metabolism was significantly reduced. This indicates a pattern of activation‐deactivation involving vestibular and visual functions during the acute phase of VN. A study using PET to measure brain metabolic changes in patients with vestibular migraine during onset and intermission periods found an increase in metabolism in the temporal–parietal lobe, insula, and bilateral thalamic regions, indicating activation of the central vestibular pathway, and a decrease in metabolism in the occipital cortex, suggesting mutual inhibition between the visual and vestibular systems (Shin et al. 2014). This decline in activity may be due to a decreased responsiveness to visual stimuli, potentially to alleviate visual oscillations (oscillopsia). Furthermore, a PET study using caloric stimulation of the vestibular apparatus revealed a significant decrease in cerebral blood flow in the bilateral occipital visual cortex, which does not depend on changes in retinal input (with eyes closed) or visual movement input, thereby protecting the organism from the effects of inappropriate visual input (visual oscillations) (Brandt et al. 1998; Wenzel et al. 1996). Stimulation of the vestibular system can cause the deactivation of the occipital cortex, which is referred to as visual–vestibular inhibitory interaction (Klingner et al. 2016). When the neural activity of the vestibular system increases, it will lead to a decrease in neural activity in the visual system, such as the occipital lobe, which is in line with the results of our study. Conversely, when the visual area is activated during walking, the vestibular area will be deactivated to protect the perception of visual self‐movement, preventing conflicts that may arise from involuntary head acceleration (Brandt 1999). This functional significance is clear in suppressing mismatches between sensory inputs by prioritizing dominant or more reliable patterns (Dieterich and Brandt 2000).

#### 4.1.2 The Spontaneous Activity Was Reduced in the Frontoparietal Lobes

Patients with BPPV exhibited a reduction in PerAF values in the right SMA, bilateral paracentral lobules, and left precuneus. It has been reported that SMA is mainly responsible for both spontaneous and triggered movements and is an active part of the sensorimotor network (SMN) (Chen et al. 2022; Zhang et al. 2017). The paracentral lobule, which has been linked to the movement of the body in space and the movement of the eyeball, along with the postcentral gyrus and precentral gyrus, collaboratively contributes to the formation of the sensory–motor cortex. Research has shown that dysfunction of the paracentral lobule can cause abnormal sensorimotor integration (Shen et al. 2022; Teggi et al. 2016; Wei et al. 2020; Zhang et al. 2017). The precuneus, meanwhile, is the parietal region that produces most vestibular responses and is related to the integration between vestibular and visual systems (Fu et al. 2022; Lopez and Cullen 2023). Klingner et al. (2013) studied the functional changes in relevant brain regions as seen by fMRI during temperature stimulation of the vestibular apparatus. They discovered that the postcentral gyrus, precentral gyrus, SMA, precuneus, and occipital lobe exhibited negative activation, suggesting that the activities of somatosensory, motor, and visual cortex were inhibited. Bense et al. (2001) observed that electrical stimulation of the vestibular apparatus could lead to the suppression of the visual and somatosensory cortex, which could be attributed to the inhibitory relationship between sensory systems; this effect is also seen between sensory and motor systems (Hlushchuk and Hari 2006). This could be a result of the nonspecific inhibition of other sensory regions during vestibular excitation. Studies have revealed that VN induces a considerable decrease in glucose metabolism in the visual and somatosensory cortex, exhibiting a strong similarity to the inhibition of the visual and somatosensory systems observed in healthy individuals during vestibular stimulation (Dieterich and Brandt 2008). Our study suggested that, in addition to the occipital visual cortex, spontaneous activity in certain regions of the frontoparietal lobe also displayed inhibitory changes, which were mainly related to the inhibitory interaction between vestibular, visual, and sensorimotor system, further indicating the nonspecific inhibitory effects of vestibular system activation on other sensory systems.

#### 4.1.3 The Spontaneous Activity Was Increased in the Cerebellar Vermis

Our research also revealed that the spontaneous activity in the brain region near the vermis was increased in individuals suffering from BPPV. Studies have identified that dizziness is associated with the activation of regions such as the insular cortex, inferior parietal lobule, putamen, cerebellum, anterior cingulate gyrus, precentral gyrus, superior temporal gyrus, and thalamus, which provides us with new knowledge about the neural basis of dizziness (Varangot‐Reille et al. 2022). The pathogenesis of BPPV involves the movement of the patient's head, which causes otolith fragments to move within the semicircular canal, disrupting lymphatic flow and causing the cupula of the ampullary crest to deviate. Alternatively, the increased density of ampullary crest cupula due to otolith fragments sedimentation can lead to deviation of the cupula with gravity, resulting in vestibulo‐ocular reflex, positional nystagmus, and dizziness in patients (Imai and Inohara 2022). The cerebellum plays a significant role in the vestibular system as an adaptive processor. The vestibulocerebellar cortex is composed of the flocculonodular lobe and the vermian cortex. The vestibulocerebellar region is directly stimulated by the peripheral vestibular receptors, such as the semicircular canal and otolith, and it regulates the vestibulo‐ocular reflex and vestibulo‐spinal reflex by sending inhibitory signals to the brainstem vestibular nucleus (Highstein and Holstein 2006; Khan and Chang 2013). An increase in ALFF and fALFF in the area near the vermis of the cerebellum in patients with BPPV suggests an adaptive change in the function of the central vestibular system. This is likely due to the repeated positional dizziness episodes during the duration of the disease, which leads to an increase in the excitatory input to the peripheral vestibular apparatus and an enhancement of spontaneous activity in the vestibulocerebellum.

### 4.2 The Connection Between the Spontaneous Activity Alterations and RD

This study discovered that patients with BPPV experienced a decrease in PerAF value in specific brain regions, including the bilateral paracentral lobules, right SMA, and left precuneus. The PerAF values in these brain regions were found to be inversely related to the severity of dizziness reported on the dizziness VAS 1 week after repositioning (W1). Fu et al. (2022) revealed that patients with BPPV with RD after repositioning had significantly lower ALFF in the bilateral precuneus compared to those without RD. Lin et al. (2023) observed a decrease in ALFF in the right precuneus indicating reduced local functional activity in RD patients. These results imply a connection between RD and functional changes in the precuneus, which is in line with our findings. Additionally, in the left fusiform and lingual gyrus of occipital regions, a negative correlation between PerAF values and DHI at baseline (W0) was observed. These findings suggest a connection between functional changes in the brain and clinical symptoms, indicating that more severe dizziness is associated with more pronounced functional changes in relevant brain regions. As previously stated, the dizziness VAS score at W1 reflects the severity of short‐term RD, and a higher DHI score at baseline is associated to the development of moderate to severe RD (Jiang et al. 2022; Wu et al. 2023). It is also hypothesized that individuals with higher DHI scores may experience a delay in central adaptation (Faralli et al. 2016). However, the specific mechanism behind this delayed central readaptation remains unclear. Roberts et al. (2018) observed in their fMRI analysis of VN patients that the BOLD signal in the V1 region exhibited a negative correlation with scores on vertigo‐related questionnaires. This indicated that individuals with the greatest reduction in BOLD signal in the primary visual cortex presented with the most severe dizziness symptoms and functional impairment. Furthermore, our study suggested that adaptive mechanisms linked to visual cortex play an important role in the central compensation for peripheral vestibular disorders. The participants in this study had suffered from BPPV for approximately 1 week, and most of them were still experiencing RD, suggesting that the central adaptation process had not yet fully taken effect. Our study suggests that functional alterations in the visual cortex and precuneus are adaptive responses associated with RD. Further studies are required to explore the relationship between functional alterations in the visual cortex or precuneus and the development of RD of patients with BPPV.

### 4.3 The Seed‐Based FC Alterations Between the Precuneus and Other Brain Regions

Our research revealed abnormal FC between various brain regions in patients with BPPV. By using different seeds from spontaneous activity analysis, such as fALFF and ALFF, we were able to identify reduced FC in specific areas of the precuneus and occipital lobes in patients with BPPV. This aligns with previous findings that have also shown changes in connectivity between the precuneus and visual cortex in individuals experiencing dizziness (Van Ombergen et al. 2017). Our findings underscore the significance of the precuneus in the integration of multiple sensory inputs in diseases related to vertigo. Because of the role of the precuneus in integrating visual and vestibular information, along with the occipital lobe's important role in visual information processing, any functional changes in these brain regions could result in abnormal integration of visual and vestibular information. In their study, Lin et al. (2023) used rs‐fMRI to discover that patients with BPPV with RD showed a decrease in FC between the right superior temporal gyrus and left precuneus. These findings may provide some explanation for the symptoms experienced by RD patients and highlight the significant role of the precuneus in the vestibular system. In their study, Li et al. (2020b) suggested that there were alterations in spontaneous functional activity and FC in the precuneus and cuneus of persistent postural‐perceptual dizziness (PPPD) patients, potentially leading to irregular integration of visual and vestibular information. Furthermore, we observed a reduction in FC between the left precuneus and paracentral lobule in patients with BPPV. The paracentral lobule is involved in controlling the movement of the body and eyeball (Teggi et al. 2016). According to Li et al. (2020a), PPPD patients showed a weakened functional connection between the precuneus and premotor cortex, which could be linked to abnormal posture control in these patients. The precuneus plays a role in integrating sensory information to create an internal estimation model. It then sends this information to the supplementary motor and premotor regions to help participate in movement and posture control (Li et al. 2020b). As a result, patients with BPPV probably display similar disturbances with integrating sensory and motor systems.

Furthermore, patients with BPPV exhibited a decrease in FC within the visual cortex located in the occipital lobe. This reduced FC is linked to the lower spontaneous activity observed in the visual cortex of patients with BPPV compared to individuals without the condition. This decrease in activity is thought to be a result of inhibitory visual–vestibular interaction, which helps to prevent conflicting sensory inputs. Si et al. (2022) found that the FC of the occipital gyrus in the visual network of patients with CUVP was significantly reduced, which was associated with a lack of visual substitution. We hypothesize that the increased excitability of the central vestibular system in patients with BPPV may lead to visual‐vestibular mismatches, causing the organism to actively avoid visual stimuli. This avoidance results in reductions in visual processing ability and FC within the visual cortex. The patients with BPPV may experience asymmetric information input from their bilateral otoliths before and after repositioning maneuvers, leading to central adaptive functional alterations. Our study indicates that the visual cortex probably plays a crucial role in this adaptive process.

### 4.4 Limitations

Certain limitations should be noted in this study. The study initially divided subjects into patients with BPPV and HCs, but the sample size was small and all subjects were from the same center. The study did not fully analyze the heterogeneity of patients with BPPV, such as the affected semicircular canals and disease course. In the future, including more subjects and further subdividing into subgroups with and without RD could help verify differences in brain function among different patient subgroups. Additionally, the average fMRI scan time for patients was approximately 2 weeks after onset, which may have impacted the results. As this study primarily used methods like ALFF, fALFF, PerAF, and FC to explore changes in brain function in patients with BPPV, it was not feasible to make causal conclusions. In the future, utilizing methods like Granger causality analysis could help to clarify the effective connectivity between functional activities in brain regions.

## 5 Conclusion

After repositioning maneuvers, certain brain regions in the bilateral occipital and frontoparietal lobes of patients with BPPV had a decreased spontaneous activity, whereas the vermis of the cerebellum had an increased spontaneous activity. FC analysis showed reductions between the left precuneus and bilateral occipital lobe, the left precuneus and left paracentral lobule and within the occipital lobes among patients with BPPV. These functional changes in the brain regions may be associated with the inhibitory interaction and functional integration of visual, vestibular, and sensorimotor systems. Additionally, the functional alterations of the precuneus and visual cortex might be relevant to RD. The outcomes of this study elucidate the modifications in brain function in patients with BPPV after repositioning maneuvers, thereby uncovering the mechanism of RD.

## Author Contributions

**Jing Wu**: software, writing—original draft, investigation, formal analysis, validation, writing—review and editing, visualization. **Liang Shu**: resources, data curation, conceptualization, methodology. **Chen‐Yan Zhou**: resources, methodology. **Xiao‐Xia Du**: supervision, investigation. **Xu‐Hong Sun**: resources. **Hui Pan**: resources. **Guo‐Hong Cui**: resources. **Jian‐Ren Liu**: supervision, funding acquisition, validation, data curation, resources. **Wei Chen**: conceptualization, methodology, resources, funding acquisition, validation, data curation.

## Ethics Statement

The Ethics Committee of Shanghai Ninth People's Hospital affiliated with Shanghai Jiao Tong University School of Medicine (ethics approval number: 2018‐128‐T106 and SH9H‐2020‐T270‐2) approved the study.

## Consent

The patients/participants provided their written informed consent to participate in this study.

## Conflicts of Interest

The authors declare no conflicts of interest.

### Peer Review

The peer review history for this article is available at https://publons.com/publon/10.1002/brb3.70053.

## Data Availability

The original contributions presented in the study are included in the article/Supporting Information, and further inquiries can be directed to the corresponding authors.
